# Bactericidal and Antiproliferative Effects of Peripheral Parenteral Nutrition Solutions with Sodium Bisulfite on Pathogenic Microorganisms in Catheter Lumens

**DOI:** 10.7150/ijms.48829

**Published:** 2020-07-11

**Authors:** Hiroshi Ohara, Masanori Watanabe, Masamu Takebayashi, Saori Abe, Tetsuya Matsuzaki, Masataka Hayasaka

**Affiliations:** 1 Department of Clinical Pharmacy, School of Pharmaceutical Sciences, Ohu University, 31-1 Misumido, Tomitamachi-Aza, Koriyama, Fukushima 963-8611, Japan.; 2 Department of Pharmacy, Ohu University Hospital, 31-1 Misumido, Tomitamachi-aza, Koriyama, Fukushima 963-8611, Japan.

**Keywords:** parenteral nutrition, sodium bisulfite, bactericidal action, microbial growth inhibition, catheter-related bloodstream infection

## Abstract

Catheter-related bloodstream infections (CRBSIs) due to pathogenic microorganisms pose a major threat to patients requiring parenteral nutrition (PN). Additives contained in medicines and foods have antiproliferative and bacteriostatic effects on pathogenic microorganisms. Therefore, PN solutions containing additives may also have an antibacterial effect. However, so far, there have been no reports on or observations of a PN solution with bactericidal activity. In this study, we assessed several nutrition solutions with antimicrobial activities and investigated their effects on pathogenic microorganisms colonizing catheter lumens. We selected the highly acidic Plas-Amino^®^ (PA), which contains a large amount of sodium bisulfite as a preservative and potentially has an antimicrobial effect. In this study, we used the following pathogenic bacteria as the main causatives of CRBSIs: *Staphylococcus aureus*, *Staphylococcus epidermidis*, *Bacillus cereus*, *Serratia marcescens*, *Pseudomonas aeruginosa*, and *Candida albicans*. We then created a catheter lumen microorganism contamination model and evaluated the antibacterial effect of PA; we found that all bacteria in the control group grew significantly in the catheter lumen in a time-dependent manner at 48 and 72 h. On the other hand, we demonstrated that PA has bactericidal effects on *S. aureus*, *S. epidermidis*, *B. cereus*,* S. marcescens*, and *P. aeruginosa* in the catheter lumen and confirmed that it has a remarkable antiproliferative effect on *C. albicans*. Hence, we concluded that highly acidic PN solutions that contain a preservative like sodium bisulfite have bactericidal and growth inhibition effects on microorganisms in the catheter lumens of patients with CRBSIs and patients with totally implantable central venous access devices, in whom it is difficult to remove the catheter.

## Introduction

Catheter-related bloodstream infections (CRBSIs) due to pathogenic microorganisms are healthcare-associated infections that result in systemic complications and have a profound effect on patient prognosis 1-6. Removal of the intravenous catheter is effective when a CRBSI occurs in a patient undergoing treatment with a nutrition solution 7. However, repeated removal and catheterization of central venous catheters (CVCs) and totally implantable central venous access devices (CVADs) in elderly patients or patients who require long-term parenteral nutrition (PN) therapy make it difficult to maintain access vessels to the central venous system and reduce the patient's quality of life. Ethanol lock therapy and antimicrobial lock therapy have been used to fight against CRBSIs in addition to catheter removal 8-13. However, the evidence and scientific basis for ethanol lock therapy are currently insufficient 7, 14 and antimicrobial lock therapy is not recommended by the Centers for Disease Control and Prevention because it may cause the pathogenic microorganisms to acquire resistance 15. On the other hand, it has been reported that taurolidine, a substance with antibacterial activity, is effective in preventing the occurrence of CRBSIs 16, 17. The European Society for Clinical Nutrition and Metabolism guidelines on home parenteral nutrition recommend taurolidine lock solution as an additional strategy to prevent CRBSIs (Grade of Recommendation B) 18. Additives or pH adjustments in pharmaceuticals or foods affect the survival of microbial pathogens. Ascorbic acid, which is widely used as a food additive and a drug, has a bactericidal effect due to the generation of reactive oxygen species resulting from the Fenton reaction 19, 20. Citric acid is also added to pharmaceuticals and foods as a pH adjuster. However, citric acid has strong acidity and antibacterial activity due to the release of hydrogen ions, which inhibit the metabolism of microorganisms 21. In addition, sodium bisulfite exerts a strong bactericidal action by inhibiting ATP synthesis and bacterial metabolism 22-24. Sodium bisulfite is used as a stabilizing agent or antioxidant in commercially available PN solutions in Japan. Therefore, in a catheter lumen contaminated with a bacterial pathogen, it is possible to sterilize it by passing a highly acidic PN solution rich in a preservative. However, the bactericidal action and growth inhibition effect of PN solutions have not yet been examined. In this study, we focused on Plas-Amino^®^ (PA) as a commercially available PN solution that contains the highest amount of sodium bisulfite as a preservative and the highest acidity in Japan 25, and investigated its effect on pathogenic microorganisms colonizing catheter lumen.

## Materials and Methods

### Microorganisms employed

Microbial pathogens were selected based on the data from a large-scale multicenter survey carried out by Wisplinghoff et al. 1 as well as the CRBSI report in Japan 26, 27. Standard American Type Culture Collection (ATCC) strains for all the microorganisms were purchased from Microbiologics, Inc. (St. Cloud, MN, USA):* Staphylococcus aureus* (ATCC6538), *Staphylococcus epidermidis* (ATCC12228), *Bacillus cereus* (ATCC11778), *Serratia marcescens* (ATCC13880), *Pseudomonas aeruginosa* (ATCC9027), and *Candida albicans* (ATCC10231).

### Test solutions

BFLUID^®^ (BF), a commercial peripheral parenteral nutrition (PPN) solution containing glucose, electrolytes, and amino acids, was purchased from Otsuka Pharmaceutical Factory, Inc. (Tokushima, Japan). Otsuka MV Injection^®^ (MVs), a commercial multivitamin preparation containing both water-soluble vitamins (vitamins B_1_, B_2_, B_6_, B_12_, and C, folic acid, nicotinamide, biotin, and panthenol) and water-insoluble vitamins (vitamins A, D, E, and K), was purchased from Otsuka Pharmaceutical Factory, Inc. In addition, PA, also purchased from Otsuka Pharmaceutical Factory, Inc., was used as a nutrition solution formulation that possibly has an antimicrobial activity. PA is a highly acidic (pH 4.6) solution that contains a higher concentration of sodium bisulfite (0.5 g/L) as a preservative than that in the other nutrition solutions 25. Tables 1 and 2 show the compositions of BF, PA, and MVs. In order to create a catheter lumen microorganism contamination model in this experiment, a test solution suitable for bacterial colonization and growth was prepared by adding MVs to 1,000 mL of BF (BF-MVs).

### Preparation of the catheter lumen microorganism contamination model, incubation, and sampling

One platinum loop from each strain was suspended and diluted in a physiological saline (Otsuka Pharmaceutical Factory, Inc.) to obtain a constant concentration of each pathogenic microorganism solution. Then, 5 mL of each solution was injected into a PLANECTA Infusion Set^®^ (JMS, Inc., Tokyo, Japan) and a TOP Extension Tube^®^ (TOP, Inc., Tokyo, Japan) connected to BF-MVs, and solution flow was blocked for 1 h. BF-MVs was added dropwise at a constant rate of 40 mL/h to remove any microorganisms that did not colonize the catheter lumen.

**Figure 1:**
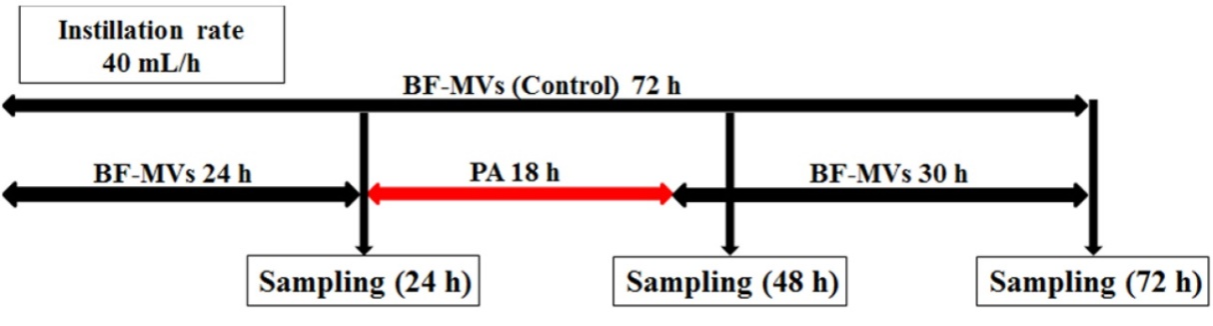
Experimental procedure, first experiment for all bacterial pathogens. In the control group, BFLUID^®^ (BF) with Otsuka MV Injection^®^ (MVs, BF-MVs) was added dropwise for 72 h. In the test group, BF-MVs was added dropwise for 24 h, and then Plas-Amino^®^ (PA) was added dropwise for 18 h, followed by the addition of BF-MVs dropwise again for 30 h. Then, after 24, 48, and 72 h, all aliquot samples were collected and the colony-forming unit (CFU)/mL value was measured.

Droplet samples (10-20 mL) were collected at 24 h after the addition of each bacterial pathogen. After sampling, we continued adding BF-MVs dropwise in the control group and replaced BF-MVs with PA in the test group for 18 h (40 mL/h). We selected 18 h because our preliminary examination showed that sterilization requires more than 12 h *in vitro* (Table 1S). Thereafter, PA was replaced with BF-MVs and added again dropwise. One aliquot (10-20 mL) of each test solution was sampled at 48 and 72 h. All experiments were performed three times at 25 °C. Figure 1 shows a schematic diagram of the experimental procedure.

### Measurement of microbial colony

All sampled test solutions were assessed using the membrane filter method in the presence of a small number of microorganisms. All aliquots (10-20 mL) were filtered using a 0.45 µm nitrocellulose membrane filter (ADVANTEC Co., Ltd., Tokyo, Japan) to perform a sterility test as described by the Japanese Pharmacopoeia 28, and then each filter was placed on a soybean-casein digest (SCD) agar medium. When necessary, any test solution sampled was serially diluted with a physiological saline before inoculation. The number of microbial pathogen colony-forming units (CFUs) on each SCD agar medium was counted after 24 h of incubation at 37 °C, and the value of CFUs/mL was calculated using the number of CFUs per SCD agar medium, the inoculum dose, and the dilution ratio. Results are represented as CFUs/mL values in semilogarithmic graphs. It has previously been shown that the biological significance of bacterial growth can be evaluated without performing any statistical analyses; hence, no statistical analyses were performed for the obtained data 29-32.

## Results

**Figure 2:**
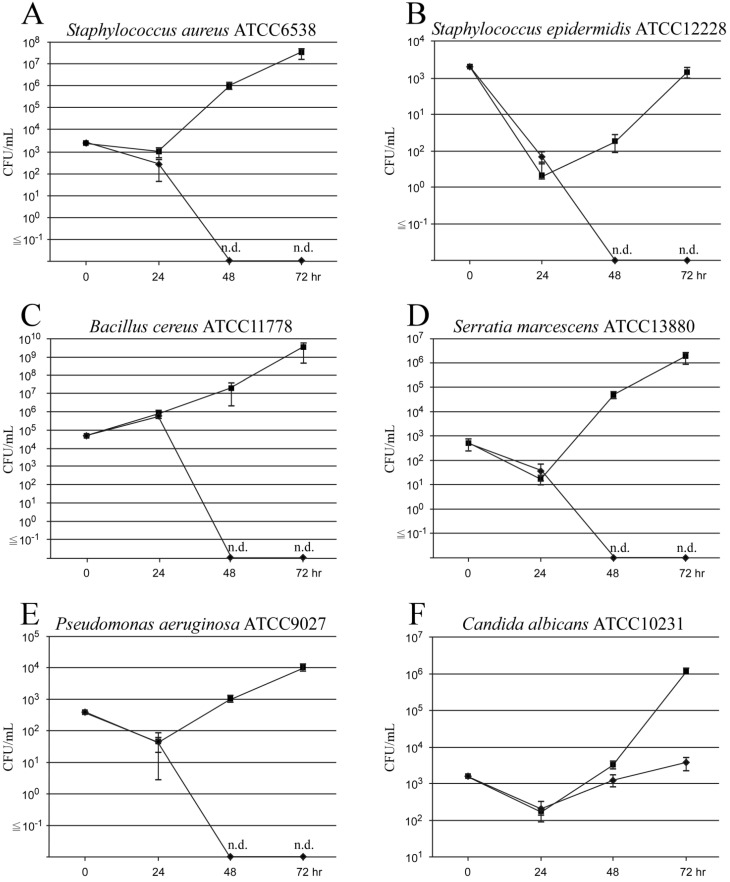
Bactericidal and antiproliferative effects of Plas-Amino^®^ (PA) on pathogenic microorganisms. Droplet samples were collected at 24, 48, and 72 h, and the colony-forming unit (CFU)/mL value was measured. (**A**) Bactericidal effect of PA on *Staphylococcus aureus*. (**B**) Bactericidal effect of PA on *Staphylococcus epidermidis*. (**C**) Bactericidal effect of PA on *Bacillus cereus*. (**D**) Bactericidal effect of PA on *Serratia marcescens*. (**E**) Bactericidal effect of PA on *Pseudomonas aeruginosa*. (**F**) Antiproliferative effect of PA on *Candida albicans*. PA (◆) and BFLUID^®^ (BF) with multivitamins (BF-MVs; ■) were used as the control. All data are represented as the mean ± standard deviation (SD; n=3). n.d.: not detectable.

In the control group (with only BF-MVs), the standard strain of *S. aureus* (ATCC6538) colonized and grew in the catheter lumen treated with BF-MVs at 24 h and rapidly increased at 48 and 72 h. In addition, *S. aureus* proliferated from 9.3 × 10^2^ CFUs/mL to 9.7 × 10^5^ CFUs/mL after 48 h and reached 3.1 × 10^7^ CFUs/mL after 72 h (Figure 2A). The standard strain of *B. cereus* (ATCC11778) significantly colonized and grew in the catheter lumen treated with BF-MVs at 24 h and rapidly increased at 48 and 72 h. Moreover, *B. cereus* proliferated from 7.1 × 10^5^ CFUs/mL to 2.0 × 10^7^ CFUs/mL after 48 h and reached 3.3 × 10^9^ CFUs/mL after 72 h (Figure 2C). However, the standard strain of *S. epidermidis* (ATCC12228) hardly colonized and grew in the catheter lumen treated with BF-MVs at 24 h and increased slowly at 48 and 72 h. Moreover, *S. epidermidis* proliferated from 2.0 × 10^0^ CFUs/mL to 1.8 × 10^1^ CFUs/mL after 48 h and to 1.4 × 10^3^ CFUs/mL after 72 h (Figure 2B). The standard strain of *S. marcescens* (ATCC13880) slightly colonized and grew in the catheter lumen treated with BF-MVs at 24 h and increased significantly at 48 and 72 h. In addition, *S. marcescens* proliferated from 1.7 × 10^1^ CFUs/mL to 4.4 × 10^4^ CFUs/mL after 48 h and to 1.7 × 10^6^ CFUs/mL after 72 h (Figure 2D). The standard strain of *P. aeruginosa* (ATCC9027) also slightly colonized and grew in the catheter lumen treated with BF-MVs at 24 h and increased slowly at 48 and 72 h. In addition, *P. aeruginosa* proliferated from 4.3 × 10^1^ CFUs/mL to 1.0 × 10^3^ CFUs/mL after 48 h and to 9.4 × 10^3^ CFUs/mL after 72 h (Figure 2E). On the other hand, no pathogenic microbes were detected after 48 and 72 h in the PA group, and it was concluded that PA has a bactericidal effect on all the pathogenic bacteria used (Figs. 2A-2E). Furthermore, the standard strain of *C. albicans* (ATCC10231) colonized and grew in the catheter lumen treated with BF-MVs at 24 h and rapidly increased at 48 and 72 h. In addition, *C. albicans* proliferated from 1.7 × 10^2^ CFUs/mL to 3.2 × 10^3^ CFUs/mL after 48 h and to 1.1 × 10^6^ CFUs/mL after 72 h (Figure 2F). Although no bactericidal effect was observed on the *C. albicans* fungus in the PA group, the addition of PA dropwise had a significant growth inhibition effect on *C. albicans* in comparison with the control group. The growth of *C. albicans* was inhibited from 2.0 × 10^2^ CFUs/mL to 1.3 × 10^3^ CFUs/mL after 48 h and to 3.7 × 10^3^ CFUs/mL after 72 h (Figure 2F).

We next performed additional experiments on *C. albicans*. A schematic diagram of the experimental procedure is shown in Figure 1S. In the first 24 h, the standard strain of *C. albicans* (ATCC10231) colonized and grew in the catheter lumen in a similar way to BF-MVs. Although no bactericidal action was observed, the intermittent administration of PA exerted a potent antiproliferative effect against *C. albicans*. The growth of *C. albicans* was inhibited from 9.0 × 10^1^ CFUs/mL to 1.8 × 10^1^ CFUs/mL after 48 h and to 2.3 × 10^2^ CFUs/mL after 72 h (Figure 2S).

## Discussion

Nutrients required for growth differ depending on the bacterial species, and there have been reports on the growth characteristics of bacteria in commercially available PPN and total parenteral nutrition (TPN) solutions 33-36. We have previously demonstrated that *C. albicans* do not grow in commercial PPN solutions but rather grow significantly in PPN solutions with biotin 37. However, much is still unknown about the relationship between bacterial growth and nutritional factors. Moreover, pH and osmotic pressure, in addition to nutrients, are important factors in the growth of bacteria. It has been shown that using a PPN solution with a high pH value and low osmolarity is more suitable for microbial growth than using a TPN solution with a low pH value and high osmolarity 38. Commercially available PN solutions contain various nutrients, making them suitable media for bacterial growth 2, 35.

We herein attempted to create a catheter lumen microorganism contamination model using typical CRBSI-causing pathogens. We added multivitamins to commercially available PPN solutions and prepared solutions (BF-MVs) more suitable for bacterial growth. When all pathogenic microorganism solutions were injected into the catheter and the flow pass was blocked for 1 h, all bacteria showed colonization and growth in the catheter lumen (Figure 2). Thus, we established a method for constructing a catheter lumen microorganism contamination model. In particular, *B. cereus* was found to colonize and proliferate significantly in the catheter lumen (Figure 2C) and to float in the air or adhere to the fingers while handling linen 26, 39. After handling linen, treatment with nutrition solutions should be avoided. Moreover, *S. aureus* were found to exhibit remarkable colonization and proliferation in the catheter lumen similar to those of *B. cereus* (Figure 2A). Shiraishi et al. reported that *S. aureus* grow in TPN solutions containing lipid emulsions 33. In addition, it has been reported that *S. aureus* require nicotinamide 36. It was also found that *S. epidermidis* and *P. aeruginosa* hardly colonize in the catheter lumen and grow slowly after 48 and 72 h (Figs. 2B and 2E). Generally, *P. aeruginosa* are resistant to a wide range of antibiotics and often cause opportunistic infections. In addition, *S. epidermidis* are considered to be the most frequently identified Gram-positive cocci and are a causative agent of CRBSIs 1. Therefore, unlike what we expected, we considered the colonization and proliferation of *S. epidermidis* in the catheter lumen to be the highest (Figure 2B), a result that is in fact very interesting. *S. epidermidis* are considered to be indigenous bacteria of the epidermis, which can enter the blood vessels during an injection and cause a BSI while using blood as a source of nutrition. It was also found that *S. marcescens* slightly colonize and proliferate in the catheter lumen and increase significantly at 48 and 72 h (Figure 2D). Omotani et al. reported that *S. marcescens* grow well in PPN solutions, which are less nutritious than TPN solutions 36. In addition, it was found that *C. albicans* proliferates rapidly 48 and 72 h after colonization in the catheter lumen (Figure 2F). Generally, CRBSIs caused by *C. albicans* are known to have a high mortality rate, and attention must be paid to the loss of vision that might probably occur because of fungal endophthalmitis 5, 40. CRBSIs are mainly caused by bacterial pathogens adhering to the catheter connection, outer circumference, and lumen during solution preparation, catheter insertion, and solution exchange 41. Our data indicate that pathogenic microorganisms can use nutrition solutions to grow when they colonize a catheter lumen. Therefore, a catheter lumen microorganism contamination model may be a useful tool to elucidate the mechanisms of CRBSIs and study how to prevent pathogenic bacterial contamination.

In this study, we investigated the effects of PA on pathogenic bacteria colonizing catheter lumens to prevent the occurrence of CRBSIs. After flowing PA into the catheter lumen contaminated with each bacterium for 18 h, PA was switched to BF-MVs and aliquots were collected 48 and 72 h later. None of the microorganisms was detected in the samples. Therefore, we concluded that PA has a bactericidal effect on *S. aureus*, *S. epidermidis*, *B. cereus*, *S. marcescens*, and *P. aeruginosa* (Figs. 2A-2E), and we confirmed that it has an antiproliferative effect on *C. albicans* (Figure 2F). In addition to chitin, *C. albicans* have a strong cell wall consisting of β-D-glucan, which promotes their survival and growth under hyperosmotic and acidic conditions, such as TPN solutions, in comparison to CRBSI-causing bacteria 34, 37. Hence, it was suggested that the penetration of sodium bisulfite contained in PA into the cells was partially blocked and that no bactericidal effect could be obtained. Among all the commercially available PPN solutions in Japan, PA is known to contain the highest concentration of sodium bisulfite (0.5 g/L) as a preservative 25. Sodium bisulfite (NaHSO_3_), which mainly exists in the form of sulfurous acid (SO_2_ or H_2_SO_3_) in its molecular form with a low pH 23, 42, 43, is a widely used preservative in pharmaceuticals and foods, which has antioxidative and antibacterial effects. This molecular form has the ability to permeate the cells and inhibit the ATP synthesis and metabolism of bacteria 22-24. Therefore, we considered PA to be highly acidic, to exhibit an increased abundance of molecular forms, and to have bactericidal and antiproliferative effects on pathogenic microorganisms. We also revealed that PA has bactericidal and antiproliferative effects on pathogenic microorganisms in catheter lumens. The limitations of this experimental model are that the treatment duration of PA was 18 h in all experiments and that we did not observe the growth trend of *C. albicans* after 72 h. Although PA is an infusion formulation that does not contain vitamins, it suffices as an energy source for the growth and development of bacterial pathogens because it mainly consists of glucose, amino acids and electrolytes. Hence, if no bacterial pathogens are detected after PA treatment, it can be concluded that the bactericidal effect observed is due to the low pH of and/or sodium bisulfite contained in PA. In this study, we found that PA remarkably suppressed the growth of *C. albicans* and demonstrated a clear bactericidal effect against bacterial pathogens other than fungi. Intermittent administration of PA against *C. albicans* may have a certain effect on the prevention of CRBSIs in cases where removing the CVC from the patient is difficult. Although catheter removal is effective in CRBSI, repeated removal and insertion can result in the loss of the catheter insertion path as a result of obstruction of the access vessel. Furthermore, implantation of totally implantable CVADs carries the risk of serious mechanical complications, such as air embolisms, arterial puncture, and pneumothorax. Intermittent administration of PA might be effective in preventing CRBSIs in cases where catheter removal from the patients is difficult. In the future, it is expected that solutions with an antimicrobial effect, such as PA, will help control CRBSIs, and we also hope that our research results prove helpful in the suppression of CRBSIs.

## Conclusion

This study shows that highly acidic parenteral nutrition (PN) solutions containing a high concentration of sodium bisulfite have bactericidal and antiproliferative effects. It was also found that Plas-Amino^®^ (PA) can be used as a peripheral parenteral nutrition (PPN) solution, which has a low risk of allergic reactions and side effects in patients. If a central venous catheter (CVC) or totally implantable CVAD becomes contaminated, it is possible to use PA to sterilize the catheter lumen and suppress bacterial growth without removing the catheter or the device. Component analysis of solutions that have secondary effects, such as PA, can help develop new lock therapy alternatives to ethanol lock therapy and antimicrobial lock therapy.

## Supplementary Material

Supplementary figures and table.

## Figures and Tables

**Table 1:** Composition of BFLUID^®^ (BF) and Plas-Amino^®^ (PA)

Category	Component	BFLUID^®^	Plas-Amino^®^
**Composition per 500 mL**			
Carbohydrate	Glucose	37.50 g	37.50 g
Amino Acids	L-Leucine	2.100 g	2.05 g
	L-Isoleucine	1.200 g	0.90 g
	L-Valine	1.200 g	1.00 g
	L-Lysine Hydrochloride	1.965 g	3.10 g
	(as L-Lysine)	(1.573 g)	(2.48 g)
	L-Threonine	0.855 g	0.90 g
	L-Tryptophan	0.300 g	0.29 g
	L-Methionine	0.585 g	1.20 g
	Acetylcysteine	0.202 g	-
	(as L-Cysteine)	(0.150 g)	-
	L-Phenylalanine	1.050 g	1.45 g
	L-Tyrosine	0.075 g	-
	L-Arginine	1.575 g	1.10 g
	L-Histidine	0.750 g	0.50 g
	L-Alanine	1.200 g	-
	L-Proline	0.750 g	-
	L-Serine	0.450 g	-
	Glycine	0.855 g	1.70 g
	L-Aspartic Acid	0.150 g	-
	L-Glutamic Acid	0.150 g	-
Electrolytes	Na^+^	17.5 mEq	17 mEq
	K^+^	10 mEq	-
	Mg^2+^	2.5 mEq	-
	Ca^2+^	2.5 mEq	-
	Cl^-^	17.5 mEq	17 mEq
	SO_4_^2-^	2.5 mEq	-
	Acetate^-^	8 mEq	-
	L-Lactate^-^	10 mEq	-
	Citrate^3-^	3 mEq	-
	P	5 mmol	-
	Zn	2.5 μmol	-
Vitamin	Thiamine Chloride Hydrochloride	0.96 mg	-
**pH**		Approximately 6.7	Approximately 4.6
**ORP**		Approximately 3	Approximately 3
**Additive Agent**	Sodium Bisulfite	0.05 g/L	0.5 g/L

ORP: osmotic pressure ratio to physiological saline.

**Table 2:** Composition of MVs (Otsuka MV Injection^®^)

Composition per 4 mL	
**Water-Soluble Vitamins**	
Vitamin B_1_	3.1 mg
Vitamin B_2_	3.6 mg
Vitamin B_6_	4.0 mg
Vitamin B_12_	0.005 mg
Vitamin C	100 mg
Folic Acid	0.4 mg
Nicotinamide	40 mg
Biotin	0.06 mg
Panthenol	14 mg
**Fat-Soluble Vitamins**	
Vitamin A Oil	3300 Vit.A IU
Vitamin D_3_	0.005 mg
Vitamin E	10 mg
Vitamin K	2 mg

Vit.A IU, international unit for vitamin A.
