# Supplementary material for: Bactericidal and Antiproliferative Effects of Peripheral Parenteral Nutrition Solutions with Sodium Bisulfite on Pathogenic Microorganisms in Catheter Lumens
Source: Int J Med Sci. 2020 Jul 11;17(12):1833–9. doi: 10.7150/ijms.48829 (PMC7378669; doi:10.7150/ijms.48829)
Supplement: Supplementary file 1 — Supplementary figures and table. [file ijmsv17p1833s1.pdf]

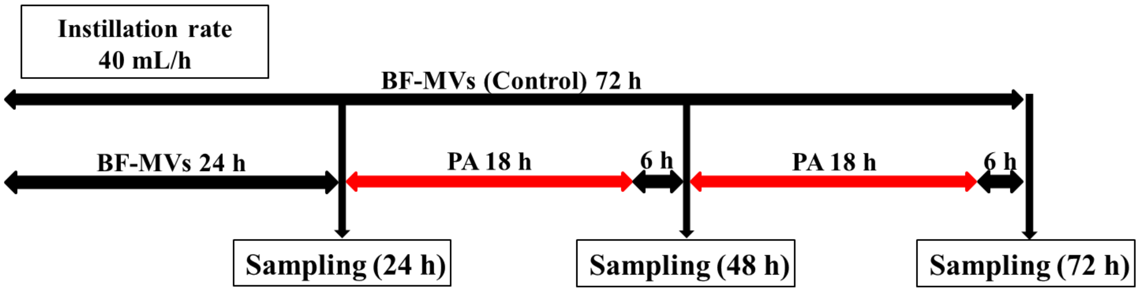

Figure 1S. Experimental procedure of the intermittent administration of Plas-Amino® (PA) into *Candida albicans*. In the control group, BF-MVs was added dropwise for 72 h. In the test group, BF-MVs was added dropwise for 24 h and then PA was added dropwise for 18 h, followed by the addition of BF-MVs dropwise again for 6 h. These steps were then repeated (intermittent administration). Then, after 24, 48, and 72 h, all aliquot samples were collected and the colony-forming unit (CFU)/mL value was measured.

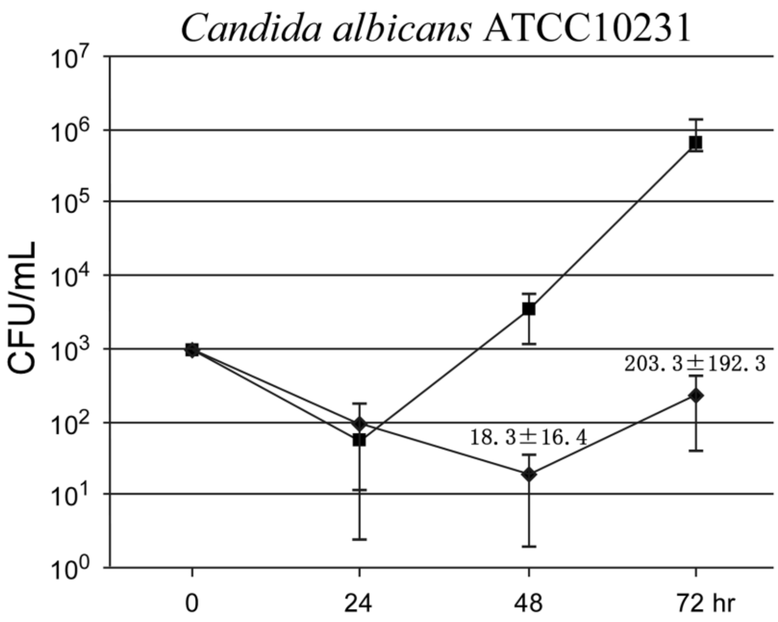

Figure 2S. Potent antiproliferative effects of the intermittent administration of Plas-Amino® (PA) on *Candida albicans*. Droplet samples were collected at 24, 48, and 72 h, and the colony-forming unit (CFU)/mL value was measured. PA (◆) and BF-MVs (■) were used as the control. Data are represented as the mean ± standard deviation (SD; $n = 3$).

Table 1S. Time course of the bactericidal effect of Plas-Amino® (PA) solutions on pathogenic microorganisms (CFUs/mL)

	**Plas-Amino® (CFU/mL)**											
	0 hr	0.25 hr	0.5 hr	1 hr	1.5 hr	2 hr	4 hr	6 hr	8 hr	10 hr	12 hr	14 hr
Staphylococcus aureus ATCC 6538	3.7×10^{2}	1.6×10^{2}	1.6×10^{2}	2.0×10^{1}	1.0×10^{1}	1.5×10^{1}	ND	ND	ND	ND	ND	ND
Bacillus cereus ATCC 11778	2.6×10^{3}	—	—	—	—	ND	ND	ND	ND	ND	ND	ND
Pseudomonas aeruginosa ATCC 9027	4.4×10^{3}	6.0×10^{3}	5.0×10^{3}	4.0×10^{3}	3.0×10^{3}	3.0×10^{3}	2.0×10^{3}	1.6×10^{3}	1.7×10^{2}	6.0×10^{1}	2.5×10^{1}	ND
Serratia marcescens ATCC 13880	4.8×10^{2}	4.5×10^{2}	5.2×10^{2}	4.8×10^{2}	3.9×10^{2}	4.1×10^{2}	4.0×10^{2}	3.7×10^{2}	2.2×10^{2}	1.0×10^{2}	4.0×10^{1}	ND

ND: not detected
